# Targeting INMT and interrupting its methylation pathway for the treatment of castration resistant prostate cancer

**DOI:** 10.1186/s13046-021-02109-z

**Published:** 2021-09-29

**Authors:** Shangwei Zhong, Ji-Hak Jeong, Changhao Huang, Xueyan Chen, Shohreh Iravani Dickinson, Jasreman Dhillon, Li Yang, Jun-Li Luo

**Affiliations:** 1 Department of Molecular Medicine, The Scripps Research Institute, Jupiter, FL 33458 USA; 2 College of Pharmacy, Research Institute of Pharmaceutical Sciences, Kyungpook National University, Daegu, 41566 Republic of Korea; 3 Department of Pathology, Moffitt Cancer Center, 2902 Magnolia Drive, Tampa, FL 33612 USA; 4 Department of Obstetrics and Gynecology, The Third Affiliated Hospital of Zhengzhou University, Zhengzhou, Henan 450052 People’s Republic of China

**Keywords:** INMT, SMYD3, Prostate cancer castration-resistance, DMT, MSA, MSC, Bis(7)-tacrine

## Abstract

**Background:**

Castration-resistant prostate cancer (CRPC) is associated with a very poor prognosis, and the treatment of which remains a serious clinical challenge.

**Methods:**

RNA-seq, qPCR, western blot and immunohistochemistry were employed to identify and confirm the high expression of indolethylamine N-methyltransferase (INMT) in CRPC and the clinical relevance. Chip assay was used to identify Histone-Lysine N-Methyltransferase (SMYD3) as a major epigenetic regulator of INMT. LC-MS/MS were used to identify new substrates of INMT methylation in CRPC tissues. Gene knockdown/overexpression, MTT and mouse cancer models were used to examine the role of INMT as well as the anticancer efficacy of INMT inhibitor N,N-dimethyltryptamine (DMT), the SMYD3 inhibitor BCl-12, the selenium compounds methaneseleninic acid (MSA) and Se-(Methyl)selenocysteine hydrochloride (MSC), and the newly identified endogenous INMT substrate Bis(7)-tacrine.

**Results:**

We found that the expression of INMT was highly increased in CRPC and was correlated with poor prognosis of clinical prostate cancer (PCa). INMT promoted PCa castration resistance via detoxification of anticancer metabolites. Knockdown of INMT or treatment with INMT inhibitor N,N-dimethyltryptamine (DMT) significantly suppressed CRPC development. Histone-Lysine N-Methyltransferase SMYD3 was a major epigenetic regulator of INMT expression, treatment with SMYD3 inhibitor BCl-121 suppressed INMT expression and inhibits CRPC development. Importantly, INMT knockdown significantly increased the anticancer effect of the exogenous selenium compounds methaneseleninic acid (MSA) and Se-(Methyl)selenocysteine hydrochloride (MSC) as well as the endogenous metabolite Bis(7)-tacrine.

**Conclusions:**

Our study suggests that INMT drives PCa castration resistance through detoxification of anticancer metabolites, targeting INMT or its regulator SMYD3 or/and its methylation metabolites represents an effective therapeutic avenue for CRPC treatment.

**Supplementary Information:**

The online version contains supplementary material available at 10.1186/s13046-021-02109-z.

## Background

Prostate cancer (PCa) is the most frequent malignancy, and the second-leading cause of cancer-related mortality in men in Western countries [1, 2]. In tumors confined to the prostate, radical prostatectomy and radiotherapy are effective, however, for late stage disseminated disease, current therapies are merely palliative [2, 3]. Androgen deprivation therapy (ADT) is a usual first-line systemic therapy for advanced PCa and is also used as an adjuvant to local therapy for high-risk diseases. Although a majority of patients initially respond to ADT, the responses in advanced disease are transient and almost all cancers eventually develop castration resistance [1–4]. Castration-resistant prostate cancer (CRPC) is associated with a very poor prognosis, and the treatment of which remains a serious clinical challenge [1–4].

Indolethylamine-*N*-methyltransferase (INMT), also named as aromatic alkylamine N-methyltransferase, indolamine N-methyltransferase, arylamine N-methyltransferase, thioether S-methyltransferase, amine N-methyltransferase, nicotine N-methyltransferase, is a methyltransferase that transfers one or more methyl groups from the methyl donor *S*-adenosyl-l-methionine (SAM) to the substrates [5–7]. INMT functions as thioether S-methyltransferase and is active with a variety of thioethers and the corresponding selenium and tellurium compounds, including 3-methylthiopropionaldehyde, dimethyl selenide, dimethyl telluride, 2-methylthioethylamine, 2-methylthioethanol, methyl-n-propyl sulfide and diethyl sulfide [8]. INMT also catalyzes the N-methylation of indoles such as tryptamine and structurally related compounds [5–7]. As indicated in its aliases, INMT also catalyzes the (N)-methylation of many other different substrates, however, these methylation substrates are largely unknown.

INMT activity is increased in schizophrenia and stress-related psychoses in humans [9]. INMT colocalizes with the sigma-1 receptors in primate spinal cord motoneurons containing unique synapses called C-terminals [10]. These findings suggest that INMT may serve as a target for the treatment of amyotrophic lateral sclerosis (ALS) and schizophrenia [9, 10]. Deregulation of INMT expression in primary cancer of lung and prostate has been reported [11, 12], however, the role of INMT in cancer is unknown.

Here, we show that INMT whose expression is highly increased in CRPC promotes PCa castration resistance via detoxification of anticancer metabolites. INMT knockdown or treatment with INMT inhibitor N,N-dimethyltryptamine (DMT) significantly suppresses CRPC development. Histone-Lysine N-Methyltransferase SMYD3 is one of the major epigenetic regulators that promote INMT expression in CRPC, treatment with SMYD3 inhibitor suppresses INMT expression and inhibits CRPC development. INMT knockdown significantly increases the anticancer efficacy of the exogenous selenium compounds methaneseleninic acid (MSA) and Se-(Methyl)selenocysteine hydrochloride (MSC) as well as Bis(7)-tacrine, an endogenous methylation metabolite substrate of INMT.

## Results

### The expression of INMT is highly increased in CRPC

**Fig. 1:**
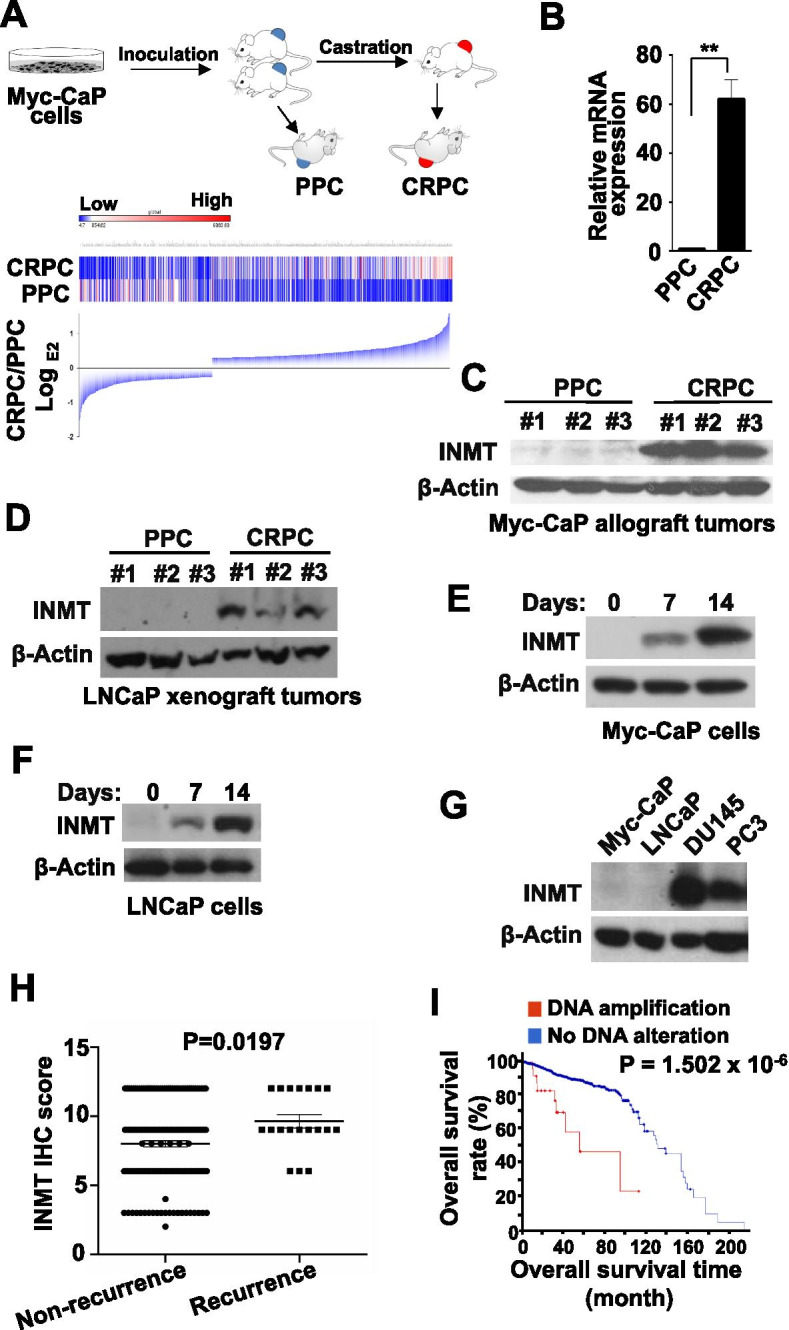
The expression of INMT is significantly increased in CRPC. **A** The diagram for different time points of allograft tumor collection in Myc-CaP CRPC mouse model (top panel), and the RNA sequencing of PPC and CRPC (bottom panel). **B** Real-time PCR analysis for INMT mRNA expression in PPC and CRPC of Myc-CaP allograft tumor tissues. **C** Western blot for INMT protein expression in PPC and CRPC of Myc-CaP allograft tumor tissues. **D** Western blot for INMT protein expression in PPC and CRPC of LNCaP xenograft tumor tissues. **E** Western blot analysis for INMT protein expression in Myc-CaP cells cultured in medium with charcoal stripped FBS for 0, 7, or 14 days. **F** Western blot analysis for INMT protein expression in LNCaP cells cultured in medium with charcoal stripped FBS for 0, 7, or 14 days. **G** Western blot for INMT protein expression in indicated PCa cell lines. **H** The expression of INMT in 192 cases of human PCa tissues detected by IHC is correlated with tumor recurrence (Non-recurrence, patients had no PCa relapse within 3 years after treatment; Recurrence, patients had PCa relapse within 3 years after treatment). The data are presented as Mean ± SEM, and the significance was calculated by the Student’s *t*-test. **I** INMT DNA amplificationin human PCa was significantly associated with patients’ survival rates. Data are from cBioPortal for Cancer Genomics database. **, *P* < 0.01

To investigate the mechanisms underlying CRPC development, we employed a prostate cancer (PCa) allograft mouse model that mimics human CRPC development [13, 14]. In this model, an androgen receptor (AR)-positive and androgen-dependent (AD) mouse prostate cancer cell line, Myc-CaP, which was isolated from a *c-Myc* transgenic mouse prostate cancer [13], was employed. Myc-CaP cells can grow as tumors in immune competent FVB male mice in an AD manner, when host mice are castrated, Myc-CaP allografts shrink, and later re-grow and become AR-positive CRPC [13, 14] (Fig. 1A).

Using RNA Sequencing and Microarray analysis, we compared the gene expressions among primary prostate cancer (PPC) and CRPC tissues, and among purified cancer cells isolated from PPC and CRPC tumors (Fig. 1A). We found that the expression of a group of genes was very significantly increased in CRPC tumors as compared with PPC tumors. INMT was one of the genes whose expressions were the most increased in CRPC tumors and purified CRPC cells. The high expression of INMT mRNA and protein in CRPC tumor tissues and in purified tumor cells was further examined and confirmed by real-time PCR (Fig. 1B), western blot analysis (Fig. 1C) and immunohistochemistry (Fig. S1A).

To exclude the possibility that the highly increased expression of INMT in CRPC is model-specific, we established another PCa xenograft mouse model, in which an AR-positive and androgen-sensitive human prostate cancer cell line, LNCaP, was employed. We found that the expression of INMT protein in LNCaP CRPC tumors was also significantly increased as compared to that in PPC tumors (Fig. 1D).

Consistently, the expression of INMT protein in both Myc-CaP and LNCaP cells was significantly increased when cultured in charcoal-treated medium for more than 7 days (Fig. 1E and F). Furthermore, the expression of INMT was significantly higher in hormone-refractory human PCa DU145 and PC3 cells, as compared with androgen-sensitive human PCa LNCaP cells cultured in normal medium (Fig. 1G). These results indicate that the expression of INMT is highly increased in CRPC and hormone-refractory PCa cells.

### The expression of INMT is associated with human PCa progression and prognosis

The expression of INMT was examined in paraffin-embedded tissue sections from 192 cases of human PCa by immunohistochemistry (IHC) (Fig. S1B), we found that the expression of INMT was significantly related to the PCa relapse rates with 3 years after treatment (Fig. 1H). Using cBioPortal for Cancer Genomics database, we mined the data related to INMT in prostate cancer. We found that the DNA amplification of INMT in PCa was also significantly associated with patients’ survival rates (Fig. 1I). These results suggest that INMT may play an important role in human PCa development and progression.

### INMT knockdown inhibits CRPC growth and development

**Fig. 2:**
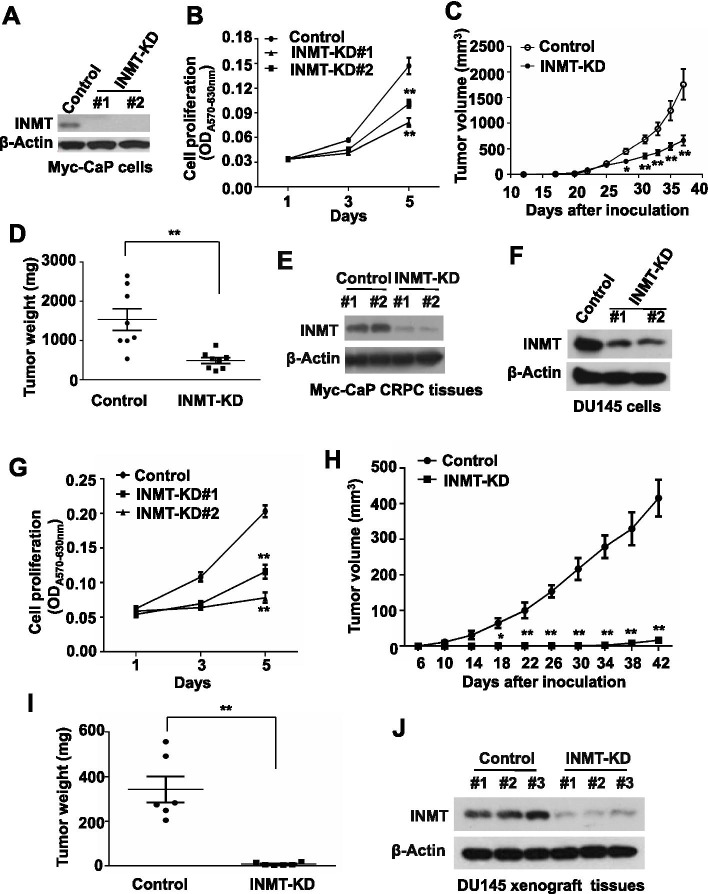
Knockdown of INMT suppresses CRPC development. **A** Western blot for the expression of indicated protein in INMT-KD and control Myc-CaP cells. **B** MTT assay for the proliferation rates of INMT-KD and control Myc-CaP cells in charcoal-treated medium. **C-E** Allograft tumor development in castrated FVB male mice inoculated with 5 × 10^5^ INMT-KD or control Myc-CaP cells (**C**). Thirty seven days later mice were sacrificed and tumor were collected and weighted (**D**), and the expression of indicated protein in tumors was analyzed by western blot (**E**). **F** Western blot for the expression of indicated protein in INMT-KD and control DU145 cells. **G** MTT assay for the proliferation rates of INMT-KD and control DU145 cells. **H-J** Xenograft tumor development in *RAG1*^−/−^ male mice inoculated with 3 × 10^6^ INMT-KD or control DU145 cells (**H**). Forty two days later mice were sacrificed and tumor were collected and weighted (**I**), and the expression of indicated protein in tumors was analyzed by western blot (**J**). *, *P* < 0.05; **, *P* < 0.01

To test the role of INMT in CRPC development, we established stable INMT knockdown (INMT-KD) cell line by infecting Myc-CaP cells with INMT shRNA lentivirus and selected by puromycin treatment. Clones with high-efficiency of INMT knockdown were confirmed by western blot (Fig. 2A) and the cell proliferation rates were examined by MTT assay. We found that although INMT-KD Myc-CaP cells had a similar proliferation rate to the control cells in normal medium (Fig. S2A), INMT-KD Myc-CaP cells grew significantly slower than control cells in charcoal-treated medium (Fig. 2B).

To examine the role of INMT in CRPC development in mouse models, INMT-KD and control Myc-CaP cells were inoculated into castrated FVB male mice, tumor development in mice was monitored (Fig. 2C). Thirty-seven days later mice were sacrificed and tumors were collected and weighted (Fig. 2D and S2B) and the knockdown of INMT in tumors was confirmed by western blot (Fig. 2E). We found that knockdown of INMT significantly decreased Myc-CaP allograft tumor development in mouse models (Fig. 2C, D, and S2B). It is worth noting that overexpression of INMT in Myc-CaP cells did not increase the proliferation of Myc-CaP cells cultured in charcoal-treated medium and Myc-CaP allograft tumor development in castrated FVB male mice (Fig. S3). These results indicate that the expression of endogenous INMT is already very high in CRPC cells, exogenous INMT expression in CRPC cells may be excess and unnecessary for the aggressive activity of CRPC cells.

To further examine the role of INMT in PCa growth, INMT was knockdown by infection with human INMT shRNA lentivirus and puromycin selection in human DU145 cells, which do not express functional AR [15, 16]. Clones with high-efficiency of INMT knockdown were confirmed by western blot (Fig. 2F) and the cell proliferation rates were examined by MTT assay. We found that knockdown of INMT in DU145 cells significantly decreased the cell proliferation rates as compared with control cells in normal medium (Fig. 2G). We also found that INMT-KD DU145 cells grew tumor much slower than control cells in *Rag1*^*−/−*^ male mice (Fig. 2H-J and S4). Together, these results suggest that INMT promotes CRPC growth and development.

### INMT inhibitor DMT shows high anticancer potency

**Fig. 3:**
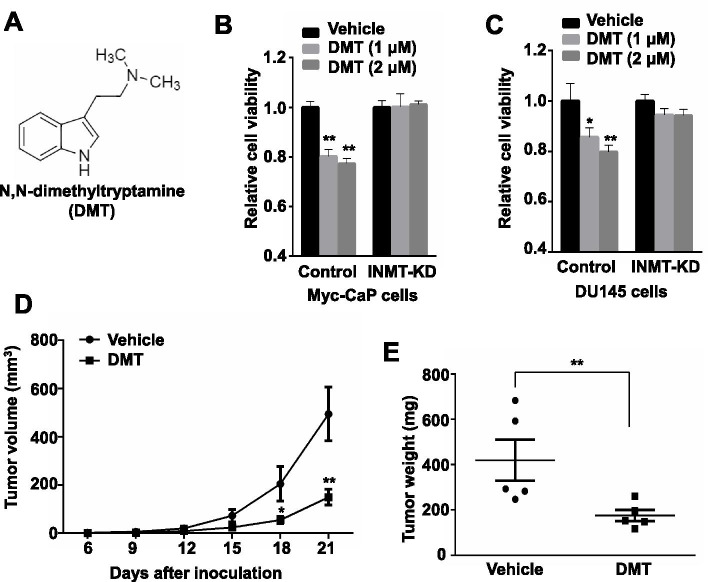
INMT inhibitor DMT suppresses CRPC growth and development. **A** The structure of DMT. **B** MTT assay for the proliferation rates of INMT-KD and control Myc-CaP cells cultured in charcoal-treated medium and treated with vehicle or DMT at different concentrations for 72 h. **C** MTT assay for the proliferation rates of INMT-KD and control DU145 cells treated with vehicle or DMT at different concentrations for 72 h. **D-E** 1 × 10^6^ Myc-CaP cells were inoculated *s.c.* into the flank of castrated FVB male mice, 3 days later followed by treatment with vehicle or DMT, *i.p*., 50 mg/kg/day (**D**). Twenty one days after cell inoculation mice were sacrificed and tumor were collected and weighted (**E**). *, *P* < 0.05; **, *P* < 0.01

It has been reported that N,N-dimethyltryptamine (DMT), a natural product of many plants [17] and being consumed as a powerful psychedelic drug [18, 19], is a mixed competitive and noncompetitive inhibitor of INMT [20] (Fig. 3A). To examine whether DMT inhibits CRPC proliferation, Myc-CaP cells were cultured in charcoal-treated medium for 3 days, followed by treatment with different concentrations of DMT and the cell proliferation rates were measured by MTT assay. We found that DMT significantly suppressed the proliferation of Myc-CaP cultured in charcoal-treated medium (Fig. 3B). Similarly, DMT also significantly inhibited the proliferation of hormone-refractory human PCa DU145 cells cultured in normal medium (Fig. 3C).

To examine whether DMT suppresses CRPC development in mouse models, Myc-CaP cells were inoculated *s.c.* into castrated male FVB mice. Three days after cancer cell inoculation, mice were divided into two groups, one group was administered *i.p.* with DMT (50 mg/kg/day), and another group treated with vehicles. We found that treatment with DMT significantly slowed down the development of castration-resistant Myc-CaP allograft tumors in FVB mice (Fig. 3D-E). Importantly, there were no apparent signs of toxicity in mice treated with DMT, as evidenced by body weight and other life symptom monitoring.

### SMYD3 is one of the major epigenetic regulators that drive INMT expression in CRPC

**Fig. 4:**
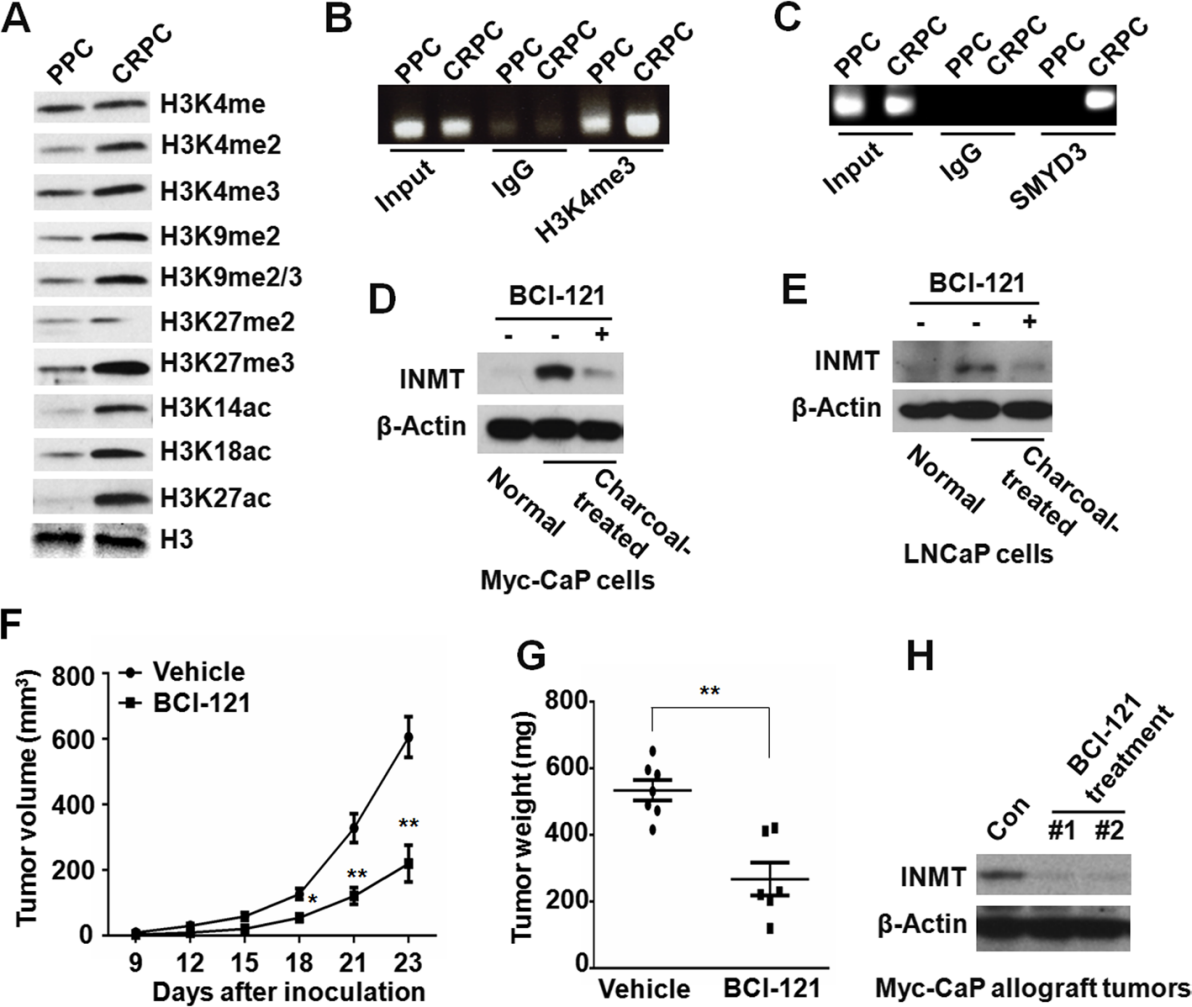
SMYD3 is one of the major regulators that drive INMT expression in CRPC. **A** Western blot for the levels of histone modification markers in purified primary PPC and CRPC cells of Myc-CaP allograft tumors. **B** ChIP assay for H3K4me3 binding to INMT promoter in purified primary PPC and CRPC cells of Myc-CaP allograft tumors. **C** ChIP assay for SMYD3 binding to INMT promoter in purified primary PPC and CRPC cells of Myc-CaP allograft tumors. **D** Western blot for INMT expression in Myc-CaP cells treated with vehicle or BCl-121 (60 μM) for 8 days in medium with normal or charcoal-treated FBS. **E** Western blot for INMT expression in LNCaP cells treated with vehicle or BCl-121 (60 μM) for 8 days in medium with normal or charcoal-treated FBS. **F-H** 1 × 10^6^ Myc-CaP cells were inoculated *s.c.* into the flank of castrated FVB male mice, 6 days later followed by treatment with vehicle or 20 mg/kg BCl-121 once a day (**F**). Twenty three days after cell inoculation mice were sacrificed and tumor were collected and weighted (**G**), and the expression of INMT in tumor tissues was detected by western blot (**H**). *, *P* < 0.05; **, *P* < 0.01

Epigenetic modification, including histone modifications, is an important regulator of gene expression [21]. Epigenetic reprogramming is closely associated with the CRPC development, and several epigenetic drugs have been developed for CRPC treatment [22–24]. To investigate the underlying mechanisms that upregulate the expression of INMT in CRPC, we compared the histone modification patterns between purified PPC cells and CRPC cells. We found that the levels of some markers were different between PPC and CRPC (Fig. 4A). To investigate whether these histone markers with different levels are related to the different expression of INMT in PPC and CRPC tumor cells, a group of anti-histone modification antibodies was used for ChIP assay. We found that the binding of H3K4me3 to INMT promoter was significantly increased in CRPC cells as compared with PPC cells (Fig. 4B). Interestingly, we found that both mouse and human *INMT* promoter had DNA binding sites (5′-CCCTCC-3′) of SMYD3 protein [25]. One of these SMYD3 binding sites was located very closely to the translational start site (ATG) of the gene (Fig. S5). Treatment with SMYD3 inhibitor BCl-121 significantly decreased INMT expression in both Myc-CaP and LNCaP cells cultured in charcoal-treated medium (Fig. 4D-E). Consistently, the expression of INMT was very low in SMYD3-KD cells as compared with that in control cells cultured in charcoal-treated medium (Fig. S6). These results showed that SMYD3 protein is a major epigenetic regulator of INMT expression.

### SMYD3 inhibitor BCl-121 suppresses CRPC growth and development

It has been reported that SMYD3 plays a role in cancer development [26, 27]. To examine the role of SMYD3 in CRPC growth and development, Myc-CaP cells were inoculated *s.c.* into castrated FVB male mice. Six days later, mice were divided into two groups, one group was administered *i.p.* with BCl-121 (20 mg/kg/day) [28], and another group treated with vehicle. We found that treatment with BCl-121 significantly slowed down Myc-CaP CRPC growth and development (Fig. 4F-G). Western blot showed that the expression of INMT in CRPC tumors from mice treated with BCl-121 was significantly decreased as compared with those treated with vehicle (Fig. 4H). These results indicate that SMYD3 inhibitor BCl-121 suppresses INMT expression and inhibits CRPC growth and development.

### INMT knockdown increases the anti-cancer efficacy of selenium compounds

INMT functions as thioether S-methyltransferase and is active with a variety of selenium compounds. Monomethylated selenium compounds have good chemopreventive and anticancer effects [29]. However, when the monomethylated selenium compounds are further methylated into dimethyl and trimethyl selenium compounds, their anticancer effect will dramatically decreased as the dimethyl and trimethyl selenium compounds are metabolized and excreted rapidly (Fig. S7A) [29].

**Fig. 5:**
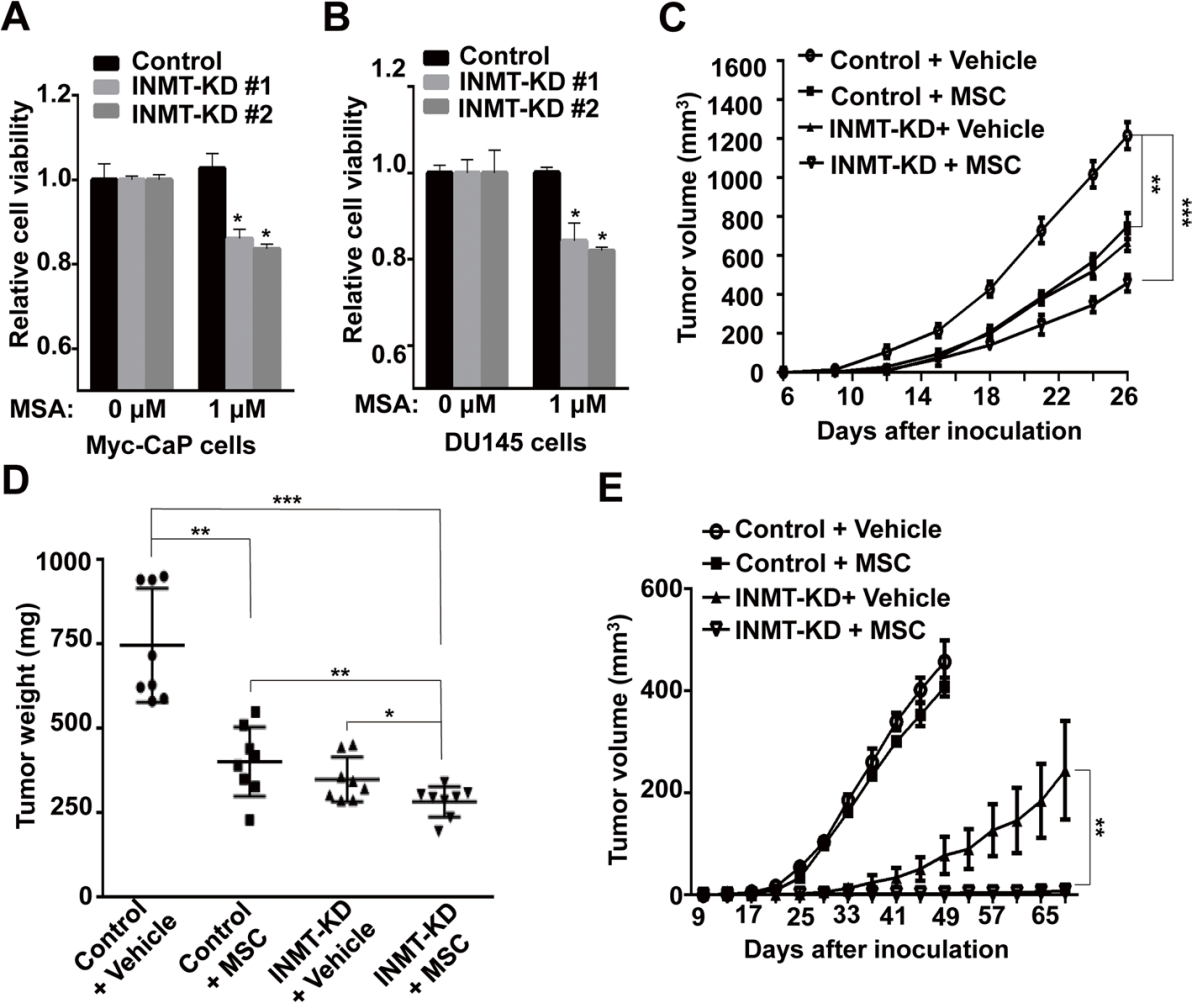
Knockdown of INMT amplifies the anticancer activity of selenium compounds MSA and MSC in vitro and in mouse models. **A** MTT analysis for the viability of INMT-KD or control Myc-CaP cells cultured in charcoal-treated medium and treated with vehicle or MSA (1 μM) for 72 h. **B** MTT analysis for the viability of INMT-KD or control DU145 cells cultured in normal medium and treated with vehicle or MSA (1 μM) for 72 h. **C, D** 1 × 10^6^ INMT-KD or control Myc-CaP cells were inoculated *s.c.* into the flank of castrated FVB male mice, 5 days later followed by treatment with *i.p.* vehicle or 5 mg/kg MSC once a day (**C**). Twenty six days after cell inoculation mice were sacrificed and tumor were collected and weighted (**D**). **E** 5 × 10^6^ INMT-KD or control DU145 cells were inoculated *s.c.* into the flank of *RAG1*^−/−^ male mice, 5 days later mice were treated with *i.p.* vehicle or 5 mg/kg MSC once a day. Tumor volume was determined at indicated intervals. *, *P* < 0.05; **, *P* < 0.01; ***, *P* < 0.001

Methylselenol is considered as the active metabolite for the anticancer effect of methyl-selenium compounds [30–32]. Two methylselenol prodrugs, methylseleninic acid (MSA) and methylselenocysteine (MSC) (Fig. S7A), are effective in suppression of (prostate) cancer cell proliferation and growth [33, 34]. As the metabolism of MSC to methylselenol requires the activity of β-lyase, which is expressed in the liver and kidney, but not the prostate cells, MSC was used only in the animal experiments. In contrast, MSA, as an oxidized form of methylselenol, is readily reduced to methylselenol through a nonenzymatic reaction in cells [35]. However, MSC showed much less toxic side effects and well tolerance [36]. Thus, in our studies MSA was used in vitro while MSC was used in animal models. We found that knockdown of INMT significantly increased the sensitivity of Myc-CaP cells to MSA in charcoal-treated medium (Fig. 5A) and the sensitivity of DU145 cells to MSA in normal medium (Fig. 5B), while overexpression of INMT resulted in Myc-CaP cells less sensitive to MSA in charcoal-treated medium (Fig. S7B). Consistently, knockdown of INMT enhanced the anticancer effect of MSC in Myc-CaP CRPC allograft (Fig. 5C-D) and DU145 xenograft (Fig. 5E) mouse models.

### Screening the endogenous substrates of INMT in CRPC

Based on the basic structures of the known substrates of INMT [5–7], we designed the experimental procedures for analyzing and comparing the potential substrates of INMT in tumor tissues. INMT-KD and control Myc-CaP castration-resistant allograft tumors were homogenized. After the protein depletion, the supernatants were transferred to HPLC vials and injected in the HPLC-MS/MS detection system. The data from HPLC-MS/MS were imported into METLIN system. We found that a group of metabolites, in which the potential (N-)methylation site(s) is unmethylated, was significantly increased in INMT-KD CRPC tumors as compared with control CRPC tumors (Table S1).

Bis(7)-Tacrine, reported as an anti-alzheimer’s and antiproliferative agent [37], was increased in INMT-KD CRPC tumors by 549 folds. Tubulosine, reported strongly inhibits HIF-1 transcriptional activity [38], was increased by 7.6 folds. 17-[(Benzylamino)methyl]estra-1,3,5(10)-triene-3,17beta-diol, whose function is unknown, was increased by 82 folds (Table S1). Importantly, some known substrates of INMT are also shown unmethylated in INMT-KD CRPC tumors, suggesting that our bioinformatics and metabolomics tools for screening INMT substrates are reliable.

### INMT knockdown enhances the anticancer activity of Bis(7)-Tacrine

**Fig. 6:**
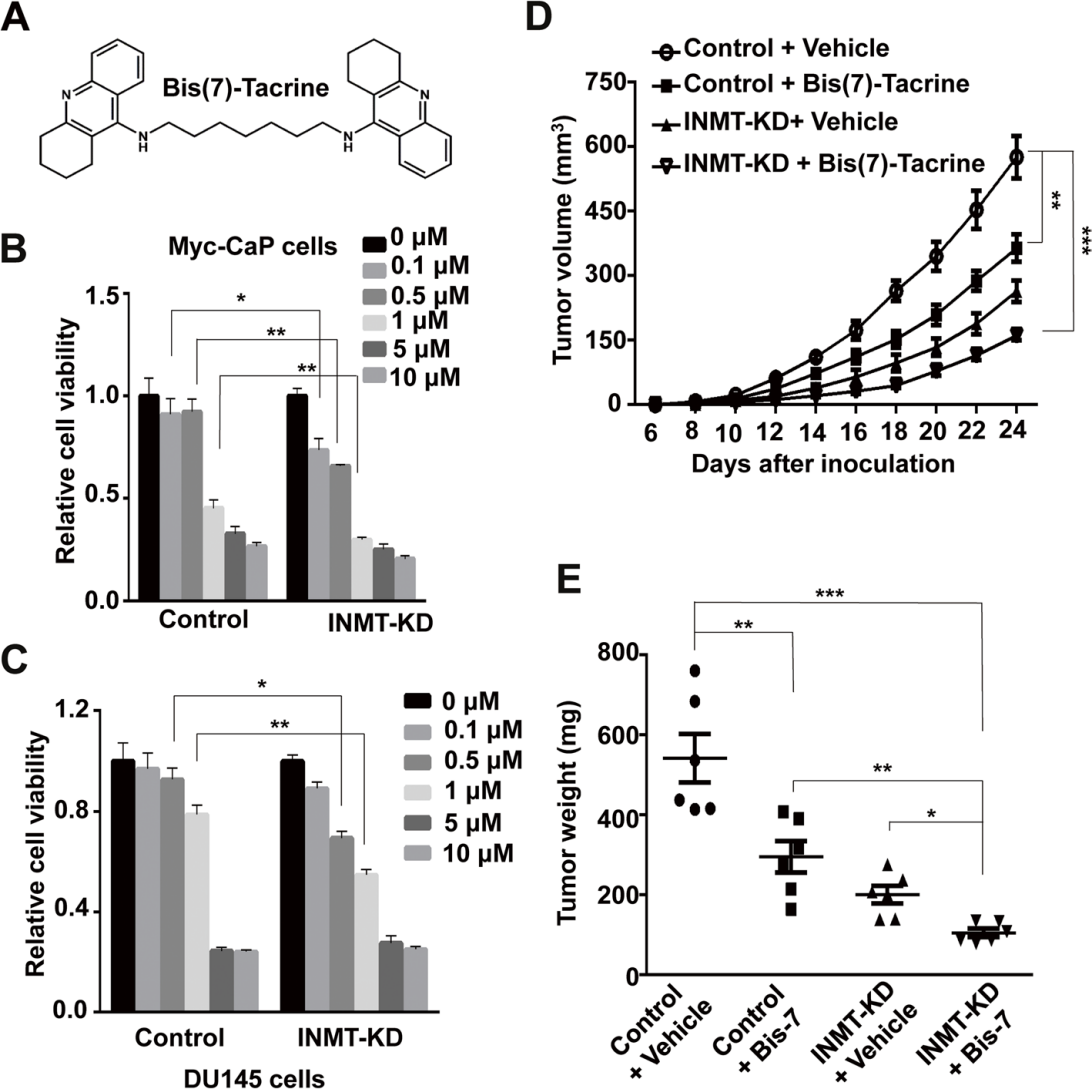
Knockdown of INMT enhances the anticancer activity of Bis(7)-Tacrine. **A** The structure of Bis(7)-Tacrine. **B** MTT analysis for the viability of INMT-KD and control Myc-CaP cells cultured in charcoal-treated medium and treated with vehicle or Bis(7)-Tacrine at different concentrations for 72 h. **C** MTT analysis for the cell viability of INMT-KD and control DU145 cells treated with vehicle or Bis(7)-Tacrine at different concentrations for 72 h. **D, E** 1 × 10^6^ INMT-KD or control Myc-CaP cells were inoculated *s.c.* into the flank of castrated FVB male mice. Five days later mice were treated with *i.v.* vehicle or 2 mg/kg Bis(7)-Tacrine once every 2 days (**D**). 24 days after cell inoculation mice were sacrificed and tumors were collected and weighted (**E**). *, *P* < 0.05; **, *P* < 0.01; ***, *P* < 0.001

As Bis(7)-tacrine was the most increased metabolite in INMT-KD CRPC tumors, the role of Bis(7)-tacrine in CRPC development was investigated. To examine whether treatment with Bis(7)-tacrine suppresses PCa androgen-independent (AI) cell proliferation, INMT-KD and control Myc-CaP cells cultured in charcoal-treated medium were treated with different concentrations of Bis(7)-tacrine for 3 days and the cell proliferation rates were examined by MTT assay. We found that although the high doses (1 ~ 10 μM) of Bis(7)-tacrine inhibited the proliferation of both control and INMT-KD cells, the low doses (0.1 ~ 0.5 μM) of Bis(7)-tacrine had much stronger effect on INMT-KD cells than control cells (Fig. 6A-B). Similarly, Bis(7)-tacrine had anti-proliferation effect on DU145 cells, knockdown of INMT significantly increased this effect (Fig. 6C). These results suggest that Bis(7)-tacrine has anti-cancer effect and INMT knockdown strongly promotes this effect.

To investigate the role of Bis(7)-tacrine in CRPC development, INMT-KD and control Myc-CaP cells were inoculated *s.c.* into right flank of castrated FVB male mice. Three days later mice were treated *i.v.* with Bis(7)-tacrine 2 mg/kg every 2 days. We found that treatment with Bis(7)-tacrine suppressed CRPC development, and INMT knockdown significantly enhanced the anticancer effect of Bis(7)-tacrine in mouse models (Fig. 6D and E). Importantly, there were no apparent signs of toxicity in mice treated with Bis(7)-tacrine.

**Fig. 7:**
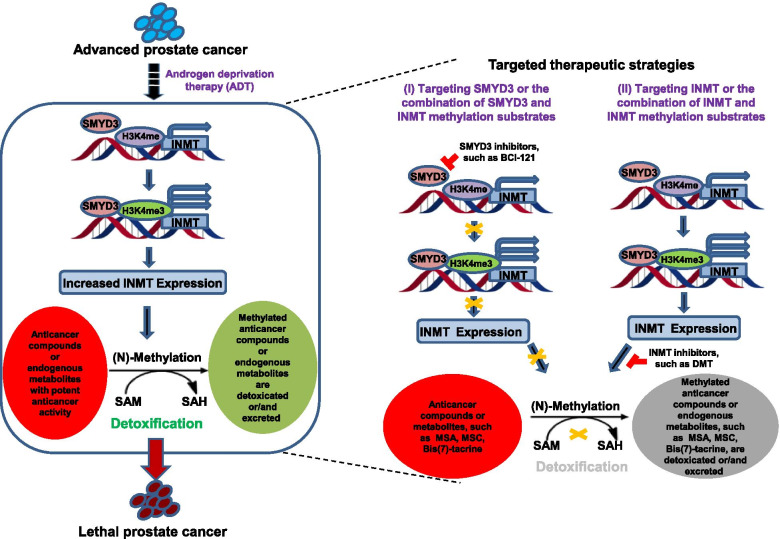
Schematic representation of the role and regulation of INMT in CRPC. The underlying mechanisms by which INMT promotes lethal prostate cancer development (left panel), and the therapeutic strategies based on targeting INMT or SMYD3 or/and the combination of INMT or SMYD3 inhibition with INMT methylation substrates (right panel)

Altogether, our studies has demonstrated that INMT, which is highly expressed in CRPC, drives CRPC growth and development. SMYD3 is one of the major epigenetic regulators that upregulate the INMT expression. INMT catalyzes the methylation and detoxification of the exogenous anticancer compounds, such as MSA and MSC, as well as the endogenous anticancer metabolites, such as Bis(7)-tacrine, resulting in PCa progression and CRPC growth and development (Fig. 7). Targeting INMT or its regulator SMYD3 or/and its methylation substrates represents an effective therapeutic avenue for CRPC treatment (Fig. 7).

## Discussion

It has been suggested that the emergence of castration resistance involves the development of cellular adaptive survival pathways in an androgen-depleted environment [1–4]. Several mechanisms for the castration resistance have been proposed, including the continuous role of AR signaling or the activation of other signaling transduction pathways in CRPC that lead to either enhanced activity of AR and its coactivators or bypassing AR in the presence of low levels or even in the absence of androgen [1, 2], the trans-differentiation of PCa stem cells into androgen-independent (AI) cell-types [1, 2], and the inflammatory signaling in both tumor microenvironment and in PCa cells that promotes the emergence of PCa castration resistance [14, 39, 40]. In present study, we demonstrate that increased expression of INMT in PCa cells drives PCa castration resistance. INMT promotes CRPC growth and development via methylation and detoxification of the anticancer metabolites that are produced in cancer cells and/or released from tumor microenvironment.

INMT is a member of a large family of *N*-methyltransferases that can methylate a variety of small molecule acceptors such as tryptamine, serotonin, and other endogenous compounds [20]. INMT activity is increased in schizophrenia and stress-related psychoses in humans [9]. INMT has been suggested as a therapeutic target for the treatment of amyotrophic lateral sclerosis (ALS) and schizophrenia [9, 10]. Deregulation of INMT expression in primary cancer of lung and prostate has been reported [11, 12], however, the role of INMT in cancer is unknown. In present study, we demonstrate that the expression of INMT is highly increased in CRPC. Knockdown of INMT as well as treatment with INMT inhibitor N,N-dimethyltryptamine (DMT) [20] significantly inhibits CRPC development in mouse models. It is worth mentioning that the finding in our report is not inconsistent with the previous report that the expression of INMT is downregulated in primary PCa [11], as our studies show INMT expression is very significantly increased in CRPC cells when compared with primary PCa cells.

INMT functions as thioether S-methyltransferase and is active with a variety of selenium compounds [8]. Methylselenol is considered as the active metabolite for the anticancer effect of methyl-selenium compounds while dimethyl selenoxide, dimethyl selenide and trimethylselenonium have no or much less anticancer activity as they are rapidly metabolized and excreted [29]. Two methylselenol prodrugs, MSA and MSC, are effective in suppression of cancer cell proliferation and growth [33, 34]. Our studies demonstrate that knockdown of INMT significantly increases the anticancer effect of MSA and MSC in vitro and in CRPC mouse models, suggesting that the combination of MSC and INMT inhibition would be an efficient way for the treatment of CRPC.

INMT catalyzes the N-methylation of indolamines, aromatic alkylamines, indolamines, amines, nicotine and structurally related compounds [5–7]. As N-methylation of endogenous and xenobiotic compounds leads to the degradation of the compounds [5], INMT may promote CRPC growth and development by detoxification of the anticancer metabolites that are produced in cancer cells and/or existed in tumor microenvironment. In present study we employ bioinformatics and metabolomics tools to screen substrate candidates of INMT in CRPC cells, and we have identified a group of metabolites that are unmethylated and increased in INMT knockdown CRPC tumors. Importantly, our studies demonstrate that treatment with one of the most increased metabolites in INMT-KD cells, Bis(7)-tacrine, significantly suppresses CRPC growth and development in vitro and in animal models. Our studies suggest that Bis(7)-tacrine and its derivatives would be potent anticancer agents for the treatment of CRPC.

Tacrine, a potent, selective and reversible acetylcholinesterase (AChE) and butyrylcholinesterase (BChE) inhibitor, approved for clinical use by the U.S. Food and Drug Administration (FDA) for the treatment of mild to moderate Alzheimer’s disease (AD) in 1993, is no longer indicated for Alzheimer’s treatment due to its hepatotoxicity [41]. However, tacrine has been used to design new non-toxic, multi-target compounds for AD, such as Bis(7)-tacrine. Compared with tacrine, Bis(7)-tacrine has few side effects, higher bioavailability, and superior efficacy [42].

Our studies also demonstrate that SET and MYND Domain containing 3 (SMYD3) is one of the major epigenetic regulators of INMT expression in CRPC. SMYD3 is a member of the lysine methyltransferase family of proteins, and plays an important role in the methylation of various histone and non-histone targets. It can specifically methylate ‘Lys-4’ of histone H3, and ‘Lys-5’ of histone H4 [25, 43]. SMYD3 can also form a complex with RNA polymerase II and bind to DNA containing 5′-CCCTCC-3′ or 5′-GAGGGG-3′ sequences presented in the promoter region that transactivates a set of downstream genes, including oncogenes, homeobox genes and genes associated with cell-cycle regulation [25]. Due to the abnormal expression of SMYD3 in tumors, it is projected as a prognostic marker in various solid cancers [25]. In the present study we demonstrate that both mouse and human INMT promoters contain SMYD3 DNA binding motif, treatment with SMYD3 inhibitor BCl-121 [28] significantly inhibits INMT expression and suppresses CRPC development in mouse models. It should be mentioned that our studies suggest that SMYD3 is the major epigenetic regulator of INMT expression in CRPC, which do not exclude the regulation of INMT expression by many other factors, such as the transcription factors.

Our studies are not only providing new insights into the molecular mechanisms underlying castration resistance, but also developing novel therapeutic strategies and new agents for the treatment of advanced PCa. The new strategies include: (1) the INMT inhibitors, for instance DMT; (2) the SMYD3 inhibitors; (3) the metabolite substrates of INMT, for instance, Bis(7)-tacrine; (4) the combination of INMT inhibition with the selenium compounds or the metabolite substrates of INMT.

Importantly, some of these INMT inhibitors and the INMT methylation substrates are currently either in clinical trials or used in clinic for noncancer diseases [18, 19]. For instance, MSC is currently in clinical trial for treatment of diffuse large B-Cell lymphoma that has relapsed or not responded to treatment (NCT00829205), and the INMT inhibitor DMT has been consumed as a powerful psychedelic drug and has historically been prepared by various cultures for ritual purposes as an entheogen [18, 19]. Thus, targeting INMT or/and its methylation substrates for the treatment of human advanced PCa is not only efficient, but also immediate practicable.

## Materials and methods

### Cell culture and reagents

Myc-CaP, an androgen sensitive mouse prostate cancer cell line, is derived from *c-Myc* transgenic mouse. LNCaP (a human androgen sensitive prostate carcinoma cell line), DU145 and PC3 cell lines are purchased from American Type Culture Collection (ATCC). These cell lines were cultured in RPMI 1640 medium supplemented with 10% fetal bovine serum (FBS) and 1% penicillin-streptomycin, and incubated in a humidified incubator with 5% CO_2_ at 37 °C. Myc-CaP cells and DU145 cells stably expressing control shRNA (Sigma), INMT shRNA (Sigma) or SMYD3 shRNA (Sigma) were cultured in RPMI 1640 medium supplemented with 10% fetal bovine serum (FBS) and puromycin (10 μg/mL). Myc-CaP cells engineered to stably express control vector and pCMV-INMT were cultured in RPMI 1640 medium supplemented with 10% fetal bovine serum (FBS) and hygromycin (100 μg/mL). DMT was purchased from Cayman Chemical company, bis(7)-Tacrine, BCl-121, Methylseleninic acid (MSA), and Se-(Methyl)selenocysteine hydrochloride (MSC) were purchased from Sigma.

### Cell proliferation assay

The CellTiter 96® Non-Radioactive Cell Proliferation Assay kit (Promega) was used to measure cell proliferation according to manufacturer’s instructions. In brief, cells were seeded into 96-well plates, after the cells cultured to indicated time-points, the MTT dye solution was added. After being incubated at 37 °C for 4 h in cell culture incubator, the Solubilization Solution/Stop Mix was added to each well, and the plates were measured at wavelength of 570 nm with 630 nm as reference wavelength using a 96-well plate reader.

### RNA isolation and real-time PCR analysis

The mRNA level of INMT was quantified using real-time polymerase chain reaction (RT-PCR) analysis. In brief, total RNA was purified from PPC and CRPC tissues using Qiagen RNeasy Mini Kit (Qiagen) according to the manufacturer’s recommendations. The cDNA was synthesized from the extracted RNA by reverse transcription using High-Capacity cDNA Reverse Transcription Kit (Thermo Fisher). Quantitative real-time PCR (qRT-PCR) was performed using SYBR Green qPCR Supermix (Solis BioDyne) according to the manufacturer’s protocol on BIO-RAD iQ5 real-time PCR Detection System (BIO-RAD). The mRNA level of INMT was normalized to the housekeeper gene GAPDH. The primers used for qRT-PCR were listed as following: mINMT-F: CCTACGACTGGTCCTCCATAGTG, mINMT-R: CTTCTGAGCTTGGCTTCCTTCT; mGAPDH-F: AGGTCGGTGTGAACGGATTTG, mGAPDH-R: TGTAGACCATGTAGTTG AGGTCA.

### Western blotting

Whole cell lysates or tissue extracts were separated by SDS-PAGE gel and then transferred to polyvinylidene fluoride membranes (PVDF) (Millipore). After being blocked in 5% nonfat milk solution for 1 h at room temperature, the membranes were incubated using specific primary antibodies for INMT (Abcam) and β-Actin (Santa Cruz) at 4 °C overnight. Followed by HRP-conjugated second antibody incubation, chemoluminescence detection was performed by using the Pierce ECL Western Blotting Reagents (Thermo Scientific).

### Chromatin immunoprecipitation (ChIP)

Chromatin immunoprecipitation (ChIP) was performed by using ChIP-IT Express kit (Active Motif) according to manufacturer’s instructions. Briefly, PPC or CRPC cells were fixed with 1% formaldehyde for 10 min, and then glycine was used to quench the reaction. Then the fixed cells were collected and suspended in ice-cold lysis buffer. Enzymatic shearing cocktail, provided with this kit, was added to shear the chromatin, then EDTA was added to stop the shearing reaction. Before the ChIP reaction with specific antibody, the sheared chromatin was pre-cleared with Protein A/G agarose beads. The supernatant was incubated with primary antibody or normal IgG overnight at 4 °C. Protein A/G agarose beads with antibody bound protein/DNA complexes were collected after centrifugation. The chromatin was eluted, reverse crosslinked, and treated with proteinase K to get DNA. PCR was performed to detect the ChIP-enriched DNA. The primers used for PCR are as following: INMT(− 127/− 11)-F: ATCTCCAGGGACCCTAGCTC, INMT(− 127/− 11)-R: CACACAGCTGTCCAGACTCC.

### Metabolism substrates of INMT analysis

To screening potentially endogenous metabolism substrates of INMT in CRPC, Myc-CaP cells stably expressing control shRNA (Control) or INMT shRNA (INMT-KD) were subcutaneously inoculated in pre-castrated FVB male mice. When the tumors reached about 500 mm^3^, the mice were euthanized and tumors were collected. 1.2 mL of cold MeOH:H_2_O (4:1, v/v) will be added into 20 mg Control or INMT-KD CRPC tissues. Then the tissues were homogenized by homogenizer, and sonicated in ice bath for 10 min. The mixtures will then be transferred to 2.0 mL Eppendorf vials and rinsed with additional 200 μL extraction solvent. To precipitate proteins, the samples were incubated for 1 h at − 20 °C, followed by 15 min centrifugation at 13,000 rpm at 4 °C. The supernatant were evaporated to dryness in a vacuum concentrator. The dry extracts were then reconstituted in ACN:H_2_O (1:1, v/v), normalized by tissue weight, sonicated for 10 min, and centrifuged 15 min at 13000 rpm and 4 °C to remove insoluble debris. The supernatants were transferred to HPLC vials for high-performance liquid chromatography (HPLC) coupled with mass spectrometry (HPLC-MS) analysis [44, 45]. The Metlin database of The Scripps Research Institute was used to search potentially endogenous metabolism substrates of INMT based on m/z value.

### Paraffin-embedded human prostate cancer samples

The study was approved by the Medical Ethics Committee of the affiliated Hospital, Zhengzhou University, and the informed consent was obtained from all the patients involved with the tissue samples. Specimens for paraffin-embed were from patients with prostate cancer who had treatment from September 2012 to 2015 at the affiliated Hospital. The clinical and pathological data of these patients were obtained from the patients’ records retrospectively.

### Immunohistochemistry (IHC)

The human prostate tumor tissue samples were stained with antibody against INMT (Invitrogen). The procedure of IHC was performed as previously described method [39]. In brief, after being treated with xylene, 100% ethanol, 95% ethanol, 80% ethanol, H_2_O, PBS, paraffin-embedded tissue samples were treated with antigen retrieval solution (Sigma). the tissue samples were incubated with the INMT primary antibody in 5% blocking serum at 4 °C overnight, after recovering 1 h at room temperature, then incubated with biotinylated secondary antibody, followed by avidin-biotinylated peroxidase complex (ABC kit) (VECTOR LABORATORIES). Finally, tissue samples were stained with DAB (3,3′-Diaminobenzidine) (VECTOR LABORATORIES) and hematoxylin. The staining results were evaluated by two independent pathologists (double-blinded) at the same time to obtain the percentage of tumor cell stained and its corresponding score (1: < 25%, 2: 25–50%, 3: > 50%) as well as the staining intensity (1-negative, 2-weak, 3-moderate, and 4-strong).The nuclear staining fraction (NF) was assigned a score of 1 (0–25%), 2 (26–50%), 3 (> 50%), and Nuclear staining intensity (NI) was noted as 1 (weak) and 2 (strong). Subsequently, a combined Nuclear score (NS) was calculated by multiplying NF and NI (range of 1 to 6).

### Animal models

FVB male mice (6 week-old) were used for mouse allograft. The mouse experimental protocols were approved by the Scripps Florida Institutional Animal Care and Use Committee and followed the guidelines of the National Institutes of Health. To investigate the expression of INMT in PPC and CRPC, 1 × 10^6^ Myc-CaP cells per point was mixed with Matrigel (Corning) and inoculated into FVB mice subcutaneously. When the tumor size reached about 500 mm^3^, some of the mice were euthanized and tumors (PPC) were collected, and the left mice were castrated, the Myc-CaP allografts shrink, and later re-grew to become CRPC, then these mice were euthanized and tumors (CRPC) were collected for further experiments. To examine the role of INMT in CRPC development, pre-castrated FVB male mice were subcutaneously inoculated with 5 × 10^5^ Myc-CaP cells stably expressing control shRNA (Control) or INMT shRNA (INMT-KD), or with Myc-CaP cells stably expressing control vector (Control) or INMT overexpression (INMT-OE), mixed in Matrigel. Rag1^−/−^ mice were used as human xenograft mouse model to examine the role of INMT in human CRPC development. Five mice for each group were included. Tumor growth was monitored and measured as indicated days.

### Quantification and statistical analysis

Differences between two groups are examined for statistical significance using Student’s *t*-test. All *p* values are two-tailed, and *p* < 0.05 was considered statistically significant (*, *p* < 0.05; **, *p* < 0.01;***, *p* < 0.001). Data are represented as means ± SEM.

## Supplementary Information

**Additional file 1.** Supplemental Information includes extending figures and is provided separately.

## Data Availability

Source data are provided with this paper. All other data supporting the findings of the study are available from the corresponding authors upon request.

## Abbreviations

INMT: Indolethylamine N-methyltransferase
CRPC: Castration-resistant prostate cancer
PCa: Prostate cancer
PPC: Primary prostate cancer
DMT: N,N-dimethyltryptamine
MSA: Methaneseleninic acid
MSC: Se-(Methyl)selenocysteine hydrochloride
ADT: Androgen deprivation therapy
SAM: S-adenosyl-l-methionine
ALS: Amyotrophic lateral sclerosis
SAH: S-adenosylhomocysteine
AR: Androgen receptor
IHC: Immunohistochemistry
SMYD3: SET and MYND Domain containing 3
RT-PCR: Real-time polymerase chain reaction
ChIP: Chromatin immunoprecipitation
HPLC-MS: High-performance liquid chromatography coupled with mass spectrometry
