# Supplementary material for: Targeting INMT and interrupting its methylation pathway for the treatment of castration resistant prostate cancer
Source: J Exp Clin Cancer Res. 2021 Sep 29;40:307. doi: 10.1186/s13046-021-02109-z (PMC8482636; doi:10.1186/s13046-021-02109-z)

Supplemental Information

**Targeting INMT and Interrupting Its Methylation Pathway for the Treatment of Castration Resistant Prostate Cancer**

**Shangwei Zhong^1^, Ji-Hak Jeong^1,2^, Changhao Huang^1^, Xueyan Chen^1^, Shohreh Iravani Dickinson^3^, Jasreman Dhillon^3^, Li Yang^1,4*^, and Jun-Li Luo^1*^**

**
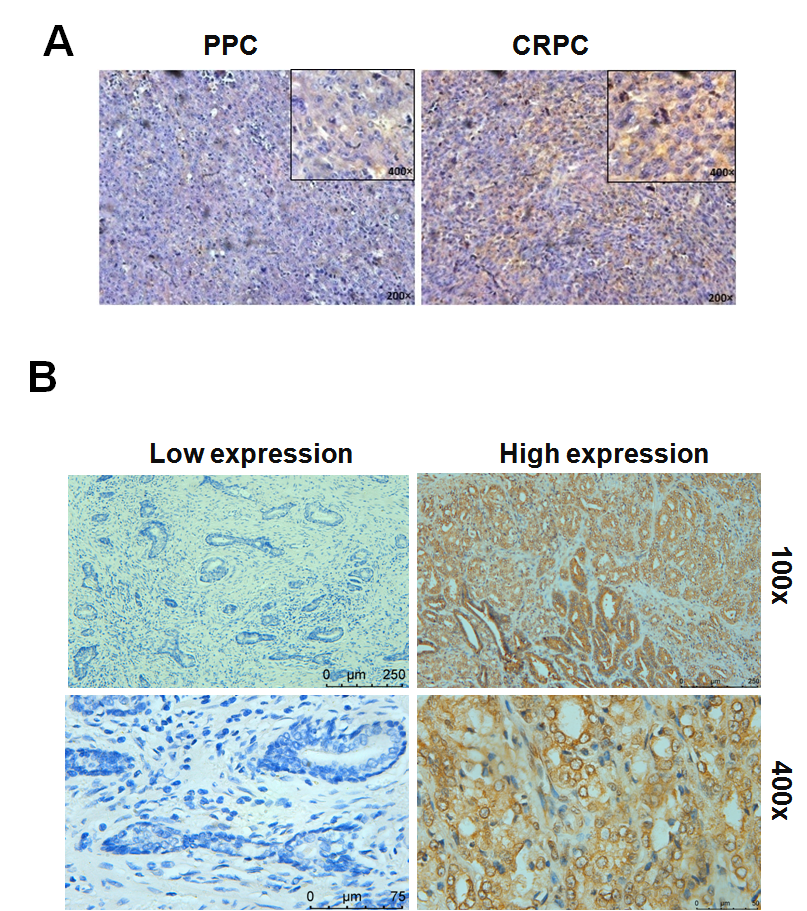
**

**Figure S1 The immunohistochemical staining of INMT in Myc-CaP allograft tumors and human PCa tissues, related to Figure 1.**

(A,B) Representative imagines of immunohistochemistry (IHC) analysis for INMT expression in paraffin-embedded Myc-CaP allograft tumor (A) and low (B-left panel) or high (B-right panel) INMT expression in human PCa tissue sections.

**
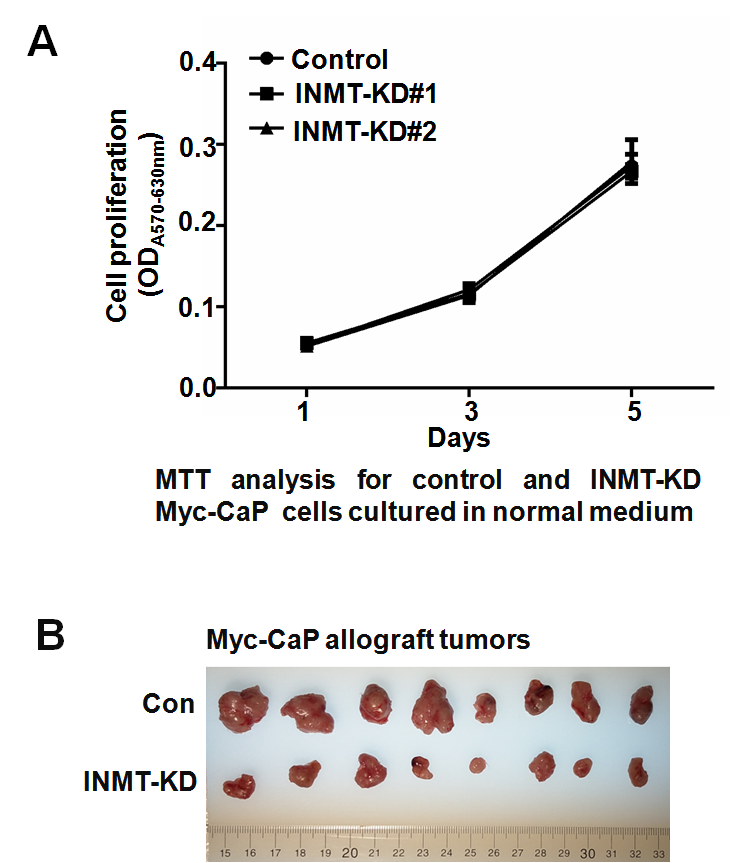
**

**Figure S2 INMT knockdown suppresses CRPC development, related to Figure 2.**

(**A**) MTT assay for the proliferation rate of INMT-KD and control Myc-CaP cells cultured in normal medium.

(**B**) The imagine of tumors collected from FVB male mice inoculated with control or INMT-KD Myc-CaP cells.

**
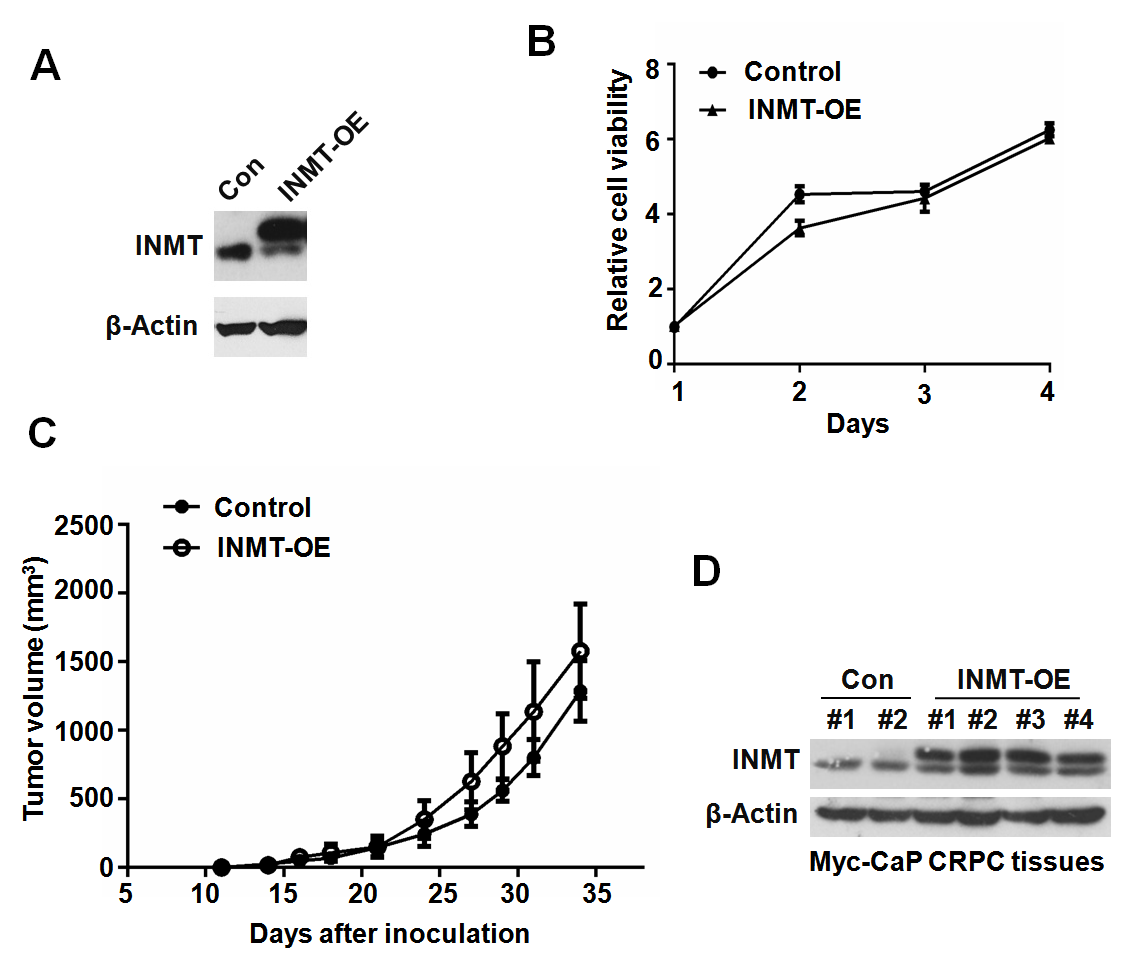
**

**Figure S3 The effect of INMT overexpression on CRPC development, related to Figure 2.**

(**A**) Western blot was performed to confirm the exogenous INMT expression (INMT-HA) in INMT-OE Myc-CaP cells.

(**B**) MTT assay for the viability of INMT-OE and control Myc-CaP cells cultured in charcoal-treated medium.

(**C**) Allograft tumor development in castrated FVB male mice inoculated with INMT-OE or control Myc-CaP cells.

(**D**) Western blot for INMT protein expression in tumors dissected from Myc-CaP allograft mouse models presented in (C).


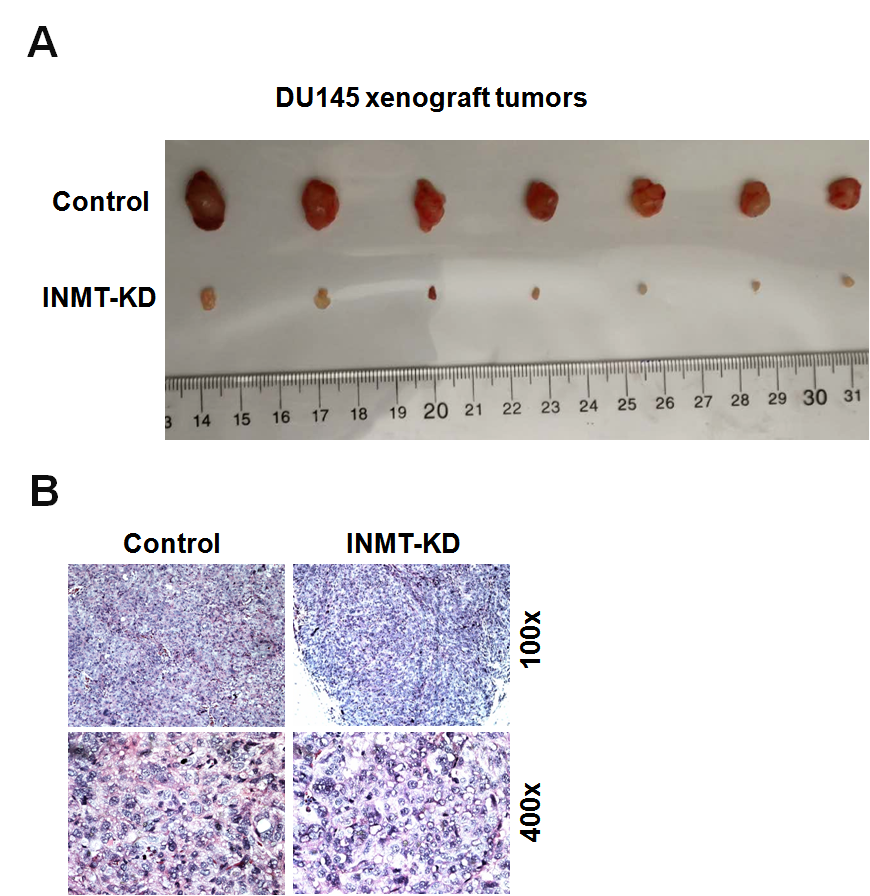


**Figure S4** The image of tumors collected from *RAG1^-/-^* male mice inoculated with control or INMT-KD DU145 cells (**A**) and the represented images of H&E staining for control or INMT-KD DU145 xenograft tumors (**B**).

**
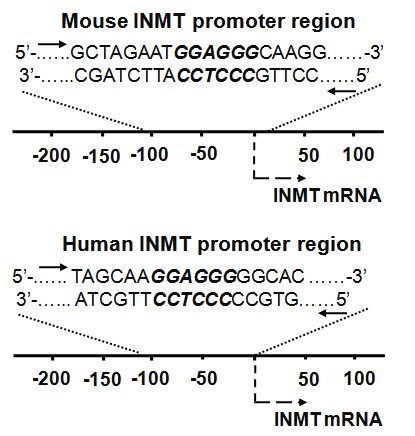
**

**Figure S5 Schematic presentation of SMYD3 protein binding site (sequences in italic) in mouse (top panel) or human (bottom panel) INMT promoter, related to Figure 4.**


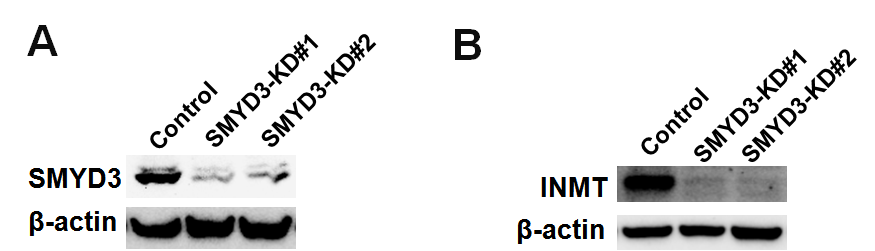


**Figure S6 The effect of SMYD3 on INMT expression**. (**A**) Western blot for the expression of indicated proteins in control and SMYD3 stable knockdown Myc-CaP cells. (**B**) Western blot for the INMT expression in control and SMYD3 knockdown Myc-CaP cells cultured in charcoal-treated medium for 7 days.

**
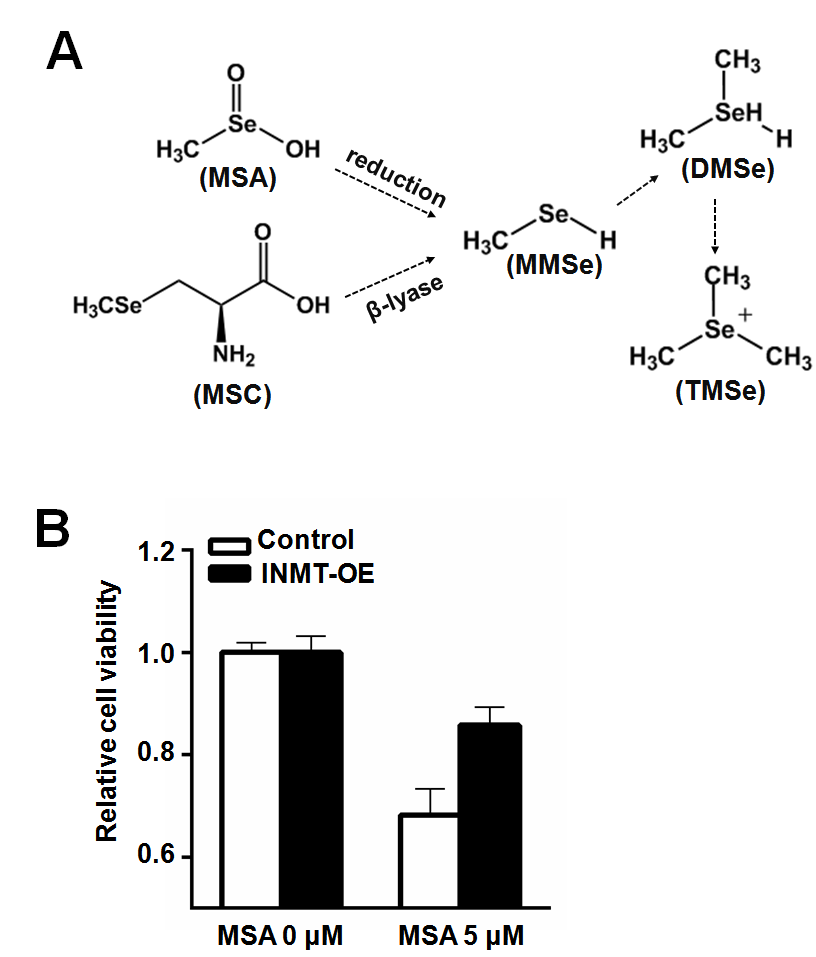
**

**Figure S7 The structure of MSA and MSC and the their anticancer activity in PCa, related to Figure 5.**

(**A**) The chemical structure of MSA and MSC, and their proposed metabolic pathway.

(**B**) MTT analysis for the viability of INMT-OE or control Myc-CaP cells cultured in charcoal-treated medium and treated with vehicle or MSA (5 μM) for 72 h.

**Table S1 Representative unmethylated substrate candidates of INMT increased in INMT-KD CRPC, related to Figure 6.**


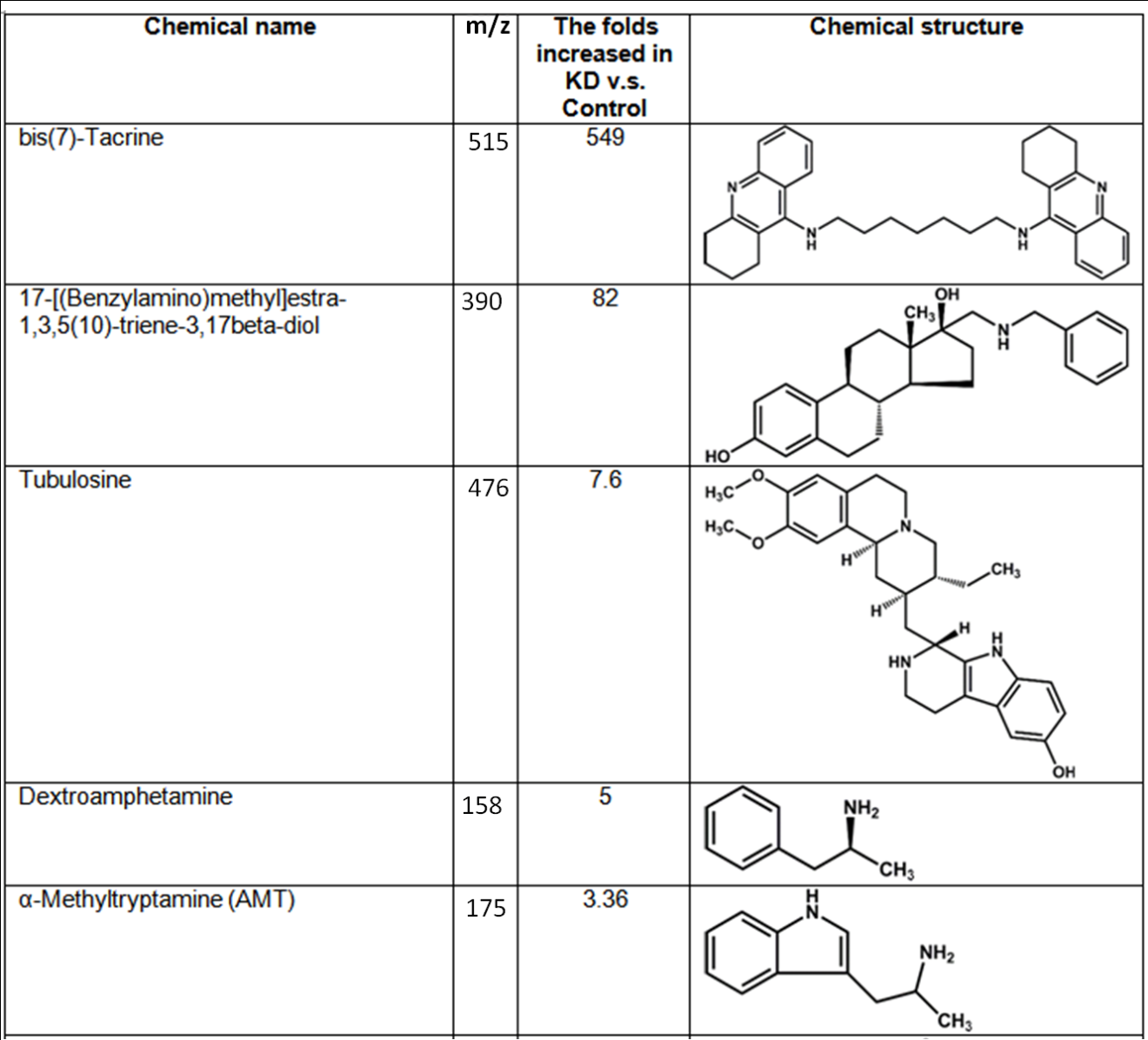

Supplement: Supplementary file 1 — Additional file 1. Supplemental Information includes extending figures and is provided separately. [file 13046_2021_2109_MOESM1_ESM.docx]
